# Tetanus Neurotoxin Neutralizing Antibodies Screened from a Human Immune scFv Antibody Phage Display Library

**DOI:** 10.3390/toxins8090266

**Published:** 2016-09-11

**Authors:** Han Wang, Rui Yu, Ting Fang, Ting Yu, Xiangyang Chi, Xiaopeng Zhang, Shuling Liu, Ling Fu, Changming Yu, Wei Chen

**Affiliations:** 1 Laboratory of Vaccine and Antibody Engineering, Beijing Institute of Biotechnology, Beijing 100071, China; happywanghan@163.com (H.W.); yurui1102@139.com (R.Y.); 15510742074@163.com (T.F.); wanghan129@sina.com (T.Y.); goodnightcxy@163.com (X.C.); xienawh@sina.com (X.Z.); 15210215727@163.com (S.L.); 15011221815@163.com (L.F.); 2 Clinical Diagnostic Centre, 302 Military Hospital of China, Beijing 100039, China

**Keywords:** tetanus neurotoxins, Hc fragment, neutralizing antibody, human immune scFv antibody phage display library, phage display

## Abstract

Tetanus neurotoxin (TeNT) produced by *Clostridium* *tetani* is one of the most poisonous protein substances. Neutralizing antibodies against TeNT can effectively prevent and cure toxicosis. Using purified Hc fragments of TeNT (TeNT-Hc) as an antigen, three specific neutralizing antibody clones recognizing different epitopes were selected from a human immune scFv antibody phage display library. The three antibodies (2-7G, 2-2D, and S-4-7H) can effectively inhibit the binding between TeNT-Hc and differentiated PC-12 cells in vitro. Moreover, 2-7G inhibited TeNT-Hc binding to the receptor via carbohydrate-binding sites of the W pocket while 2-2D and S-4-7H inhibited binding of the R pocket. Although no single mAb completely protected mice from the toxin, they could both prolong survival when challenged with 20 LD_50_s (50% of the lethal dose) of TeNT. When used together, the mAbs completely neutralized 1000 LD_50_s/mg Ab, indicating their high neutralizing potency in vivo. Antibodies recognizing different carbohydrate-binding pockets could have higher synergistic toxin neutralization activities than those that recognize the same pockets. These results could lead to further production of neutralizing antibody drugs against TeNT and indicate that using TeNT-Hc as an antigen for screening human antibodies for TeNT intoxication therapy from human immune antibody library was convenient and effective.

## 1. Introduction

Tetanus is an acute, often fatal disease of humans caused by the tetanus neurotoxin (TeNT) produced by the bacterium *Clostridium tetani*. Moreover, it is characterized by generalized rigidity and convulsive spasms of the skeletal muscles. The muscle stiffness usually involves lockjaw, the neck, and then becomes generalized. Hippocrates first described tetanus over 24 centuries ago [1]. However, as one of the most potent toxins known, even today it causes paralytic death to hundreds of thousands of humans annually [2]. As of December 2007, there were 47 countries remaining that had not eliminated neonatal tetanus (MNT) [3].

The general principles for the treatment of tetanus are supportive, such as providing an artificial respirator, as well as passively giving human Tetanus Immunoglobulin (TIG) or antitoxins containing polyclonal antibodies (PAb) derived from an animal (e.g., horse) which had been immunized with an adjuvant tetanus toxoid [4]. Due to the cost of TIG, only the horse products are available in most developing countries (e.g., Egypt) [5]. Human anti-TeNT serum from immunized volunteers has limitations related to their small-scale production and the risk of infectious disease transmission. However, the use of antitoxin from no homologous species can generate side effects, including the risk of zoonosis and anaphylactic shock [6].

A fully human specific antibody to TeNT, which can be produced in vitro without the immunization process, would be a better alternative to human anti-TeNT serum or antitoxins with large quantities and few side effects to humans [7,8]. Phage display technology is a tractable, economical, and rapid method to obtain human antibodies, in which a single-chain variable fragment (scFv) or antigen-binding fragment (Fab) antibody is expressed as a fusion with a coating protein on the surface of the phage [9].

To date, several large human phage libraries have been reported, however, seldom regarding immune antibody libraries have been published, including that for neutralizing antibodies of Tetanus neurotoxin [10,11,12]. 

Compared with the yield generated using hybridoma technology, additional antibodies can be derived from a recombinant immune library made with the material of a single immunized donor, and in vitro selection can enrich for rare antibody specificities. Furthermore, human immune or disease-associated antibody libraries have identified antibodies with very interesting properties unlikely to be present in nonimmune or synthetic libraries [13]. 

In our previous work, a human immune scFv antibody phage display library consisting of 1 × 10^8^ individual clones with high diversity had been generated and used to screen scFvs against the HC chain of TeNT (TeNT-Hc) [14]. To construct the library, V_H_ and V_L_ genes were amplified from peripheral blood lymphocytes of five volunteers who have high antibody titers against tetanus toxoid. 

The correct choice of antigen is very important in screening specific neutralizing TeNT antibodies from a phage display antibody library. TeNT is produced as a ~150-kDa protein that is cleaved to a di-chain protein, comprising an *N*-terminal light chain (TeNT-L, ~50 kDa) and a *C*-terminal heavy chain domain (H, ~100 kDa) linked through a single disulfide bond [15]. TeNT light chain is a zinc metalloprotease that cleaves the neuronal SNARE protein VAMP2 [16]. The TeNT heavy chain contains two functional domains: a translocation domain (TeNT-HN, ~50 kDa) and a *C*-terminal receptor binding domain (TeNT-Hc, ~50 kDa). TeNT-L specificity of polyclonal and monoclonal antibodies may have a crucial role in full protection against tetanus toxin. However, the mechanism by which zinc metalloprotease activity of TeNT light chain is neutralized by antibodies is not fully understood. The enzymatic groove of holo-TeNT is masked. The enzymatic active site becomes un-masked after the TeNT light chain exits from endosome into the neuronal cell cytoplasm. The antibodies with TeNT protease inhibitory activity should be developed further into a non-toxic cell penetrating antibody (transbody) [6].

TeNT-Hc is involved in binding to cellular receptors with completely non-toxic effects, and can induce protective antibodies in animals, which is considered to be a potential candidate for tetanus toxoid subunit vaccines [17,18,19,20]. The TeNT-Hc has been proved as the protective antigen and immunized with TeNT-Hc alone can achieve similar immune effect with full length tetanus toxoid does. Tetanus toxoid (TT) could induce the production of antibodies not only against TeNT-Hc but also against TeNT-HN and TeNT-L. However, the antibodies against TeNT-Hc induced by TT are the main neutralizing antibodies for protection. 

Previously, we found that a soluble form of TeNT-Hc was expressed in *Escherichia coli*, and approximately 333 mg/L (6.66 μmol/L) was acquired. The NIH mice vaccinated with TeNT-Hc adsorbed an aluminum hydroxide gel adjuvant and demonstrated a potency of 168 IU/mL, which is two times higher than the national standard for tetanus vaccines [21]. 

The purpose of this study was to screen for specific neutralizing antibodies against TeNT from the human immune scFv antibody phage display library using purified TeNT-Hc and TeNT as the antigen. Three specific antibody clones with neutralizing activities were obtained, and the mixture of the three antibodies exhibited potent toxin-neutralizing properties of at least 20 LD50s/20 µg antibody mixture. The results indicated that using TeNT-Hc and TeNT as antigens to screen human antibodies for TeNT intoxication therapy from a human immunized antibody library was convenient and effective.

## 2. Results

### 2.1. Panning and Screening of scFv Libraries

Using purified TeNT-Hc and pure active toxin as an antigen, three rounds of selection were performed. To obtain more specific and high-affinity clones in the second round, we reduced the concentration of coated antigen from 200 nM to 20 nM for purified TeNT-Hc (66.7 nM to 6.7 nM for pure active toxin), and we reduced it to 10 nM for purified TeNT-Hc (3.3 nM for pure active toxin) in the third round. After three rounds of selection, the ratio of output to input titers increased, which, in the third round, was approximately 170~220 times that of the first round. 

After three rounds of selection, the proportion of positive clones determined via ELISA was about 90%. The specificity of each TeNT-Hc scFv was determined by an ELISA for different antigens, including TeNT-Hc, F1, V, IFN-ω, and BSA. We selected two subunit vaccines protein or glycoprotein as negative antigens, *Yersinia pestis* capsular F1 and V protein, which have similar functions with TeNT-Hc. The *Yersinia pestis* virulence protein V antigen, a 37 kDa protein, was also proven to be best in eliciting protection against plague in different animal models. LcrV is a key component of the type IIII secretion system in *Y. pestis* and is required for delivery of effector proteins into host cells [22]. The *Yersinia pestis* F1 capsular antigen, a 15.5 kDa glycoprotein, forms a large gel-like capsule or envelope. It has been implicated to be involved in the ability of *Y. pestis* to prevent uptake by macrophages and is a highly protective antigen and is considered as a key constituent of a subunit anti-plague vaccine [23,24]. We also selected a 19.9 kDa cytokines, Interferon-Omega (IFN-ω), serving as different antigens in ELISA, which was secreted from cells in response to viral infection and displays antiviral, antiproliferative and immunomodulatory activities [25]. All those three antigens including *Yersinia pestis* capsular F1 and V protein, IFN-ω, were all purified in our lab.

**Figure 1:**
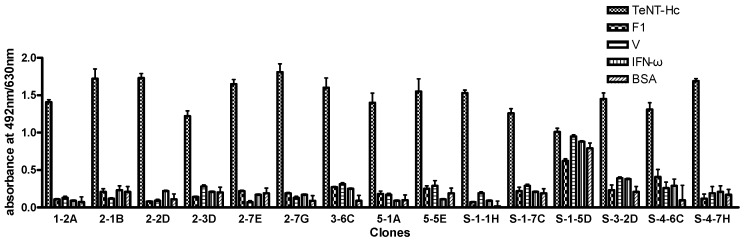
ELISA characterization of a partial phage-scFv clone binding to different proteins. Assays were performed by immobilizing TeNT-Hc, F1, V, IFN-ω, and BSA-coated on a polystyrene plate. Phage-scFvs derived from the library that were reactive with the coated antigen, were detected with a 1:5000 dilution of horseradish peroxidase (HRP)–conjugated anti-M13 antibody. The results of the assay are shown as the absorbance at 492 nm/630 nm. Assays were performed in triplicate, and the range is shown.

Approximately 85% of the clones could specially bind to TeNT-Hc, with no cross-reactivity to other proteins (Figure 1). A total of 12 clones were selected based on the high OD values (OD > 1.5) that were sent for sequencing analysis. Eight different sequences were identified from the 12 clones that were sequenced.

### 2.2. Secreted Expression of scFvs by E. coli

The genes of eight unique scFvs that were bound to TeNT-Hc were digested and inserted into the *E. coli* secretion vector, kil-SN for secreted expression. In the presence of the colicin release protein kil, scFvs can be easily released from the periplasm into the growth medium (Figure S1). The scFvs were purified (Figure S2), and their specific binding to TeNT-Hc was determined by an ELISA, which indicated that all eight expressed scFvs maintained the specificity of binding to TeNT-Hc (Figure S3).

### 2.3. Characterization of TeNT-Hc Specific scFvs

Since the epitope of the TeNT-Hc antibody was essential to its neutralizing activity, and the combination of antibodies mapping different epitopes could increase their neutralizing potency, a competitive binding ELISA was performed to search for specific antibodies mapping different epitopes. The results (Table 1) revealed that any of the paired clones of 2-1B, 3-6C, 2-7G, 5-1A, 5-5E, and S-1-1H could completely bind to TeNT-Hc, and the inhibition efficiency was from 53% to 87%, suggesting that the six clones mapped the same or similar epitopes. 2-2D and S-4-7H could not be inhibited by any clone of 2-1B, 3-6C, 2-7G, 5-1A, 5-5E, or S-1-1H, suggesting that they mapped different epitopes from others. Among the clones that mapped the same epitope, the inhibition potency of 2-7G to 2-1B, 3-6C, 5-1A, 5-5E, and S-1-1H was the highest, which may be due to the high affinity of 2-7G. In the former selections, we still obtained many specific clones that had not been sequenced. A competitive binding ELISA was also used to detect the epitopes of 2-2D and S-4-7H. The result showed that 2-2D and S-4-7H could not be inhibited by each other. 

**Figure 2:**
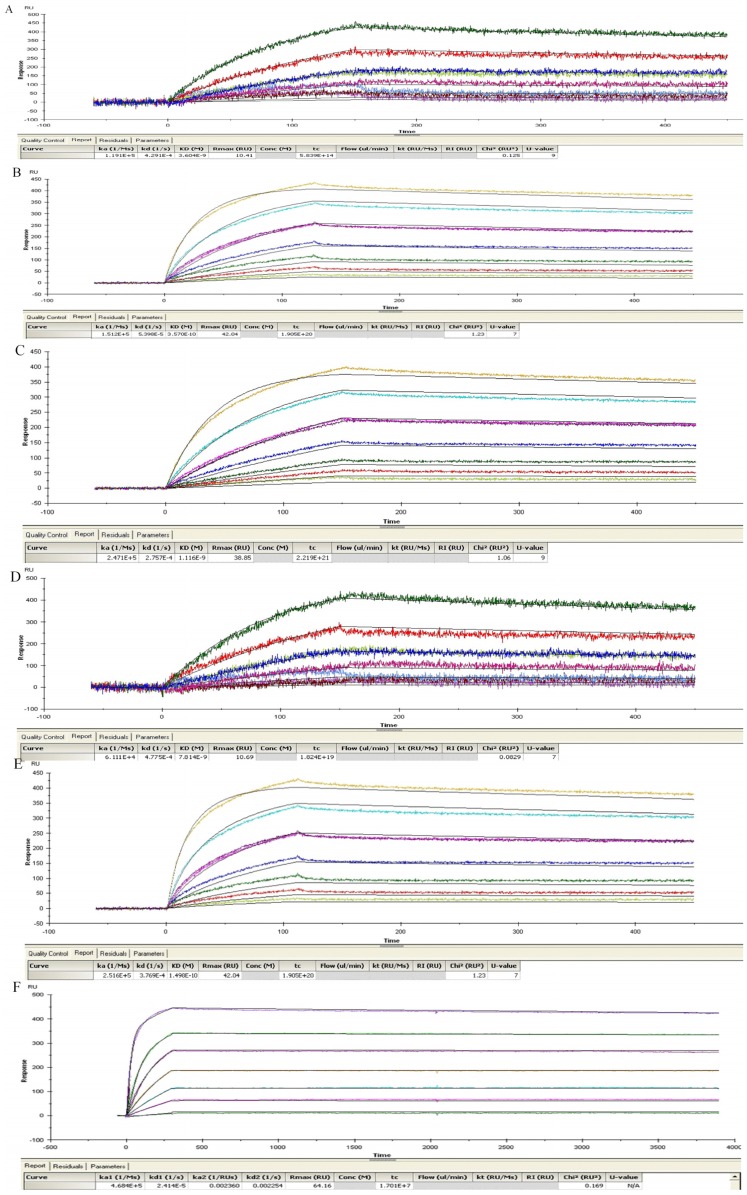
Affinities and binding kinetics of scFvs and lgGs binding TeNT. Binding curve of scFvs with TeNT, obtained using both Biacore T200. The curve was globally fit to yield equilibrium dissociation constants (KD). The equilibrium dissociation constant for each antibody-antigen pair is computed as KD = koff/kon. The results are listed in Table 3 and Table 4. (**A**,**D**,**E**) Binding curves of S-4-7H, 2-2D and 2-7G of the scFv with TeNT; (**B**,**C**,**F**) Binding curves of S-4-7H, 2-2D and 2-7G of the IgG with TeNT.

The affinities of 2-7G, 2-2D, and S-4-7H bound TeNT were determined by surface plasmon resonance (SPR) spectroscopy (Figure 2). 2-7G, 2-2D, and S-4-7H antibodies with a KD of 1.50 × 10^−10^ M, 7.82 × 10^−9^ M and 2.25 × 10^−9^ M were obtained (Table 2). The three antibody clones can easily be associated with the antigen and are difficult to dissociate. The sequencing results demonstrated that the V_L_ genes of 2-7G and 2-2D belonged to the V_κ3_ gene family, and the V_H_ genes of 2-7G and 2-2D belonged to V_H3_ gene family. The V_L_ genes of S-4-7F belonged to the V_L1_ gene family, and its V_H_ genes belonged to V_H6_ (Table 3). Families of heavy and light chain and the CDR3-Sequences of the eight selected clones were showed as supplemental data (Tables S1 and S2).

### 2.4. Construction and Expression of IgG4 Antibodies

**Figure 3:**
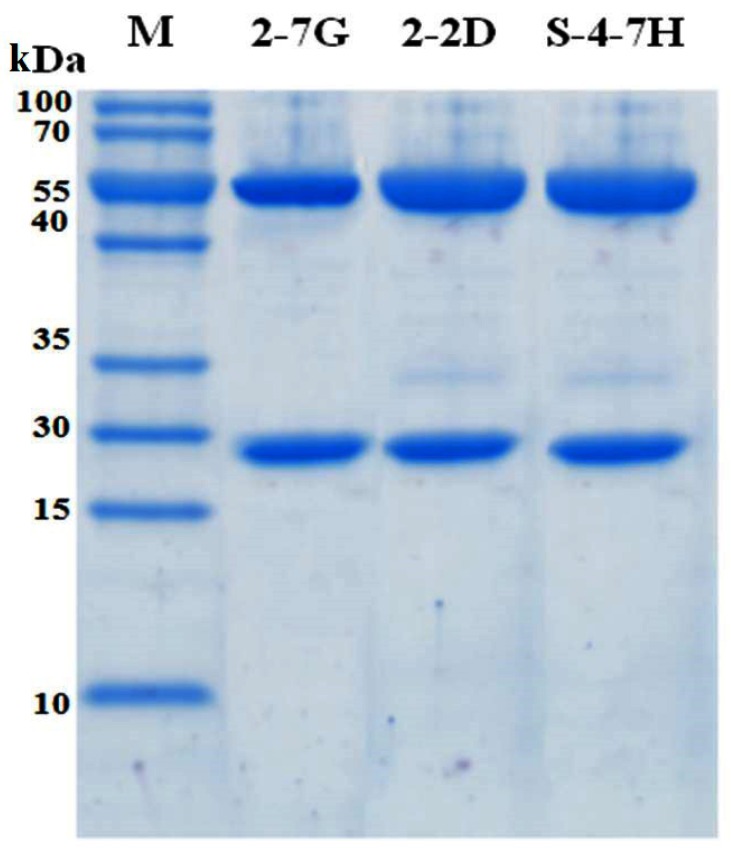
The 12% sodium dodecyl sulfate-polyacrylamide gel electrophoresis (SDS-PAGE) analysis (reducing) of purified 2-7G, 2-2D, and S-4-7H IgG4.

Due to the rapid clearance of scFv from serum, in vivo toxin neutralization activity might not be determined precisely. To establish more stable antibodies, 2-7G, 2-2D, and S-4-7H IgG4 were constructed by inserting the V_H_ and V_L_ genes into the mammalian expression vectors H293 and L293-C λ, resulting in the fusion of V_H_ and V_L_ genes to C_H_ and C_L_ genes. IgG4 antibodies could barely activate the classic pathway of complement, and thus the neutralizing antibodies could not be cleared immediately by macrophages in vivo. The plasmids were transfected into FreeStyle^TM^ 293-F cells for instantaneous expression. The FreeStyle^TM^ 293 Expression System was designed to allow for the large-scale transfection of suspension 293 human embryonic kidney cells in a defined, serum-free medium. The IgG4 antibodies of 2-7G, 2-2D, and S-4-7H yielded high expression levels in this system, and were purified on a Protein A column. The purified protein contained a 50-kDa heavy chain and a 28-kDa light chain, which corresponded with the entire antibody (Figure 3). Since IgGs were glycosylated and scFv from *E. coli* not, we measured the three IgGs with TeNT by SPR. The antigen binding affinity of 2-7G, 2-2D, and S-4-7H of the IgG was significantly higher (lower KD) than for the corresponding scFv. 2-7G, 2-2D, and S-4-7H antibodies with a KD of 5.20 × 10^−11^ M, 1.12 × 10^−9^ M and 3.57 × 10^−10^ M were obtained (Table 4).

### 2.5. In Vitro Neutralizing Potency of Antibodies against TeNT

**Figure 4:**
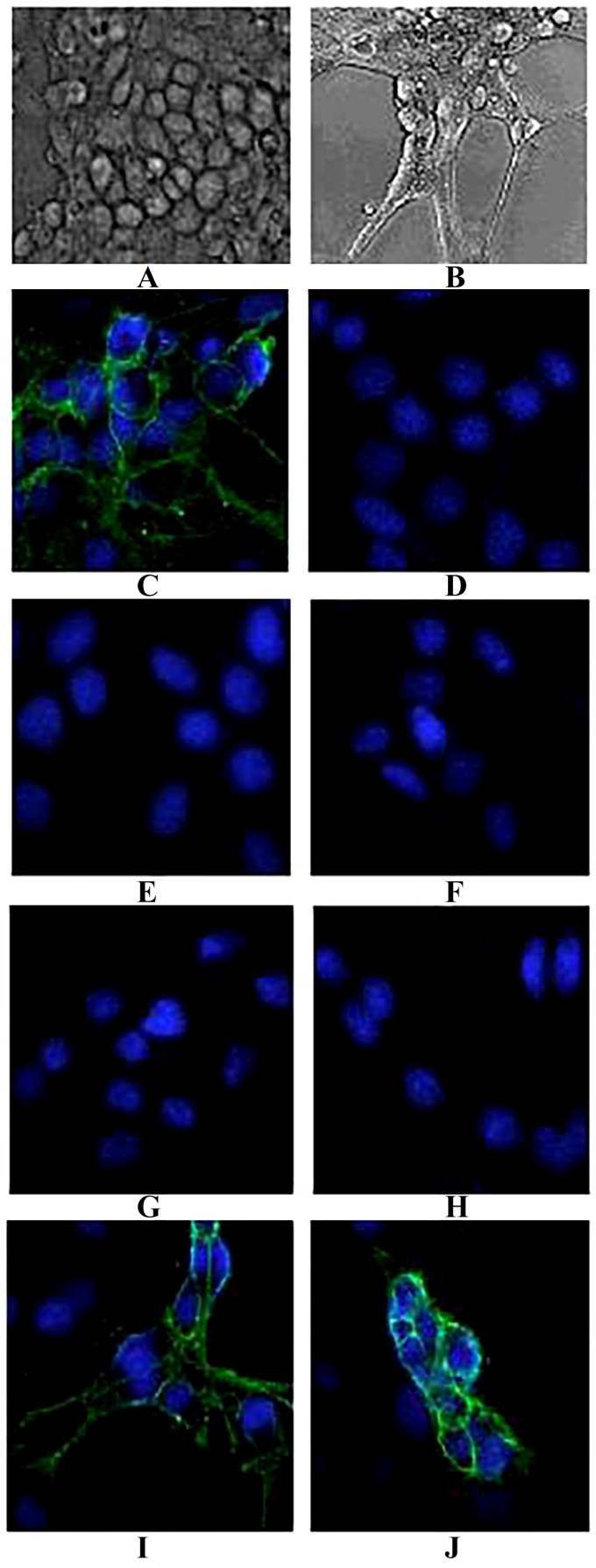
The inhibition of 2-7G, 2-2D, and S-4-7H IgG4 antibodies via the interaction between TeNT-Hc and NGF–treated PC-12 cells detected by a cellular immunofluorescence assay. (**A**)Phase contrast images of prefixed PC-12 cells; (**B**) Phase contrast images of NGF-treated PC-12 cells; (**C**) TeNT-Hc was incubated with prefixed PC-12 cells and detected by the mouse polyclonal anti-TeNT-Hc antibodies followed by FITC labeled anti-mouse IgG antibodies. The lighter fluorescence surrounding the cell membranes suggested that TeNT-Hc could bind to PC-12 cells; (**D**) Bovine serum albumin (BSA) was used instead of TeNT-Hc and detected by the mouse monoclonal anti-BSA antibodies, and no binding to PC-12 cells was observed; (**E**) The mouse polyclonal anti-TeNT-Hc antibodies were used as a positive control, which could effectively inhibit the interaction between PC-12 cells and TeNT-Hc in vitro; (**F**) 2-7G IgG4 antibodies were mixed with TeNT-Hc before incubating with the PC-12 cells, and no binding between TeNT-Hc and PC-12 cells was detected; (**G**) 2-2D IgG4 antibodies were mixed with TeNT-Hc before incubating with the PC-12 cells, and no binding between TeNT-Hc and PC-12 was observed; (**H**) S-4-7H IgG4 antibodies were mixed with TeNT-Hc before incubating with the PC-12 cells, and no binding between TeNT-Hc and PC-12 was found; (**I**) An anti–*Yersinia pestis* capsular F1 protein (F1) antibody was used as a negative control, which could not inhibit the interaction between TeNT-Hc and PC-12 cells; (**J**) An anti–*Yersinia pestis* capsular V protein (V) antibody was used as a negative control, which could not inhibit the interaction between the TeNT-Hc and PC-12 cells. The magnification of the cells is 200 times.

Tetanus neurotoxins can bind to various types of cells, including the NGF-treated neuronal-like PC-12 cells through the Hc domain. NGF can induce the differentiation of PC-12 cells which develop extended neuritis and an increased number of synaptic-like vesicles (Figure 4B). First, the binding ability of TeNT-Hc to PC-12 cells was determined by a cellular immunofluorescence assay, and the results showed that TeNT-Hc could bind to the membrane of PC-12 cells (Figure 4C), whereas BSA could not (Figure 4D). When antibodies were cultured with TeNT-Hc before being added to the PC12 cells, 2-7G, 2-2D, and S-4-7H could effectively inhibit TeNT-Hc binding to PC-12, similar to the mouse polyclonal anti-TeNT-Hc antibodies (Figure 4E–H). An anti–*Yersinia pestis* capsular F1 and V protein antibody could not inhibit TeNT-Hc binding to PC-12 (Figure 4I–J).

### 2.6. Inhibition of TeNT-Hc Binding to Gangliosides by 2-7G, 2-2D, and S-4-7H via Two Carbohydrate-Binding Sites

Representative curves are shown here and the average inhibition percentage of TeNT-Hc binding to gangliosides by mAbs is calculated. Each point is the average of three wells. An increasing concentration of TeNT-Hc (expressed by our laboratory, from 6 nM to 400 nM) in the presence or absence of appropriate concentration (66.7 nM) of mAbs was allowed to bind to ganglioside GT1b/GM1a/GD3. Percent inhibition of TeNT-Hc binding was calculated by dividing of average OD of each concentration of neutralizing mAbs by the average OD of toxin alone. The data was plotted using GraphPad Prism version 5 (2007, GraphPad Software Inc., 7825 Fay Avenue, Suite 230 La Jolla, CA, USA). Immobilized ganglioside added with TeNT-Hc mixed with the PBS (without Abs) served as negative inhibition control. The percentages of Abs mediated inhibition of TeNT-Hc binding to gangliosides calculated. % binding inhibition was 100% - % TeNT-Hc binding to gangliosides.

**Figure 5:**
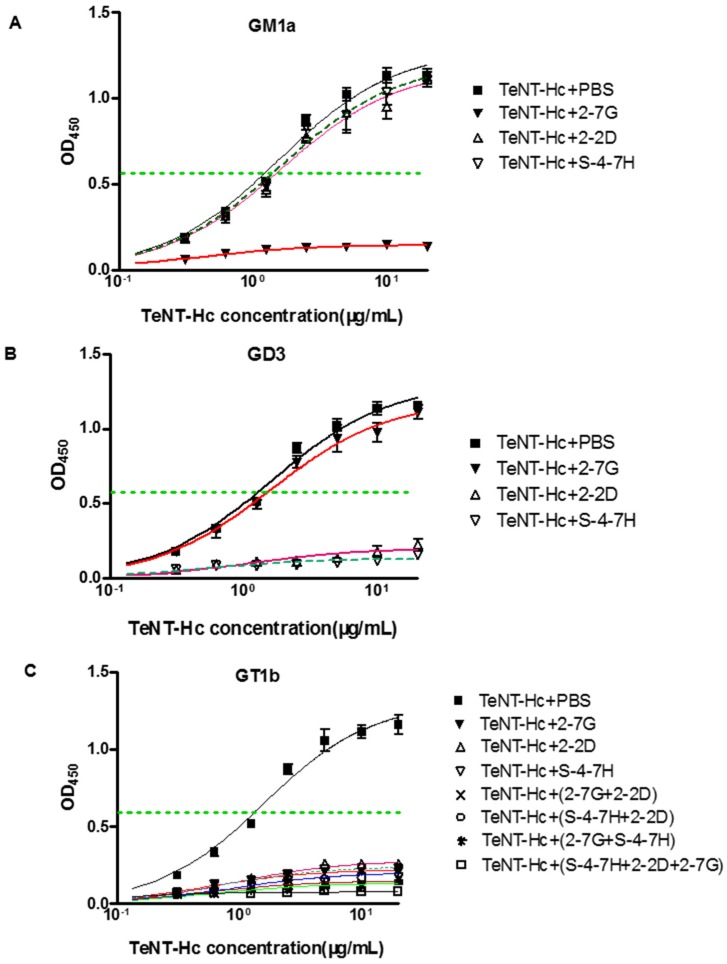
Inhibition of TeNT-Hc binding to gangliosides by 2-7G, 2-2D, and S-4-7H antibodies (Ab). (**A**) The ability of Ab-mediated inhibition of TeNT-Hc binding to GM1a; (**B**) The capacity of Ab-mediated inhibition of TeNT-Hc binding to GD3; (**C**) The ability of Ab-mediated inhibition of the TeNT-Hc binding to GT1b. The dotted lines represented the half-maximal ELISA signal of TeNT-Hc binding to ganglioside.

For determining the Ab-mediated inhibition of the TeNT-Hc binding to the receptor-dependent carbohydrate-binding site, three different assays were performed. The ability of Ab-mediated inhibition of the TeNT-Hc binding to GT1b, GM1a, and GD3 were analyzed individually (Figure 5). It was found that, for GM1a, in the presence of 2-7G, 2-2D, and S-4-7H, the percentage binding of the TeNT-Hc was reduced to 7.3%, 98.3%, and 97.7%, respectively, when compared to the binding of TeNT-Hc in PBS (absent of Abs), which was regarded as 100% binding (Figure 5A). Thus, the binding inhibitory activity of 2-7G, 2-2D, and S-4-7H was 92.7%, 1.7%, and 2.3%, respectively. It was also shown that there was a 2-7G inhibition of TeNT-Hc binding to the receptor via the carbohydrate-binding sites of the W pocket in which the toxin binding domain of the 2-7G epitopes belong. For GD3, the binding inhibitory activity of 2-7G, 2-2D, and S-4-7H was 2.5%, 85.6%, and 88.1%, respectively. This suggested that the 2-2D and S-4-7H inhibited the binding of TeNT-Hc to the receptor via the carbohydrate-binding sites of the R pocket (Figure 5B). For GT1b, the binding inhibitory activity of 2-7G, 2-2D, S-4-7H, and a various combination of the three Abs ranged from 78.6% to 99.6%. The inhibitory activities for the combination of the Abs were higher for the Ab alone, and the greatest inhibitory activity was demonstrated in the group for the combination of the three Abs combined (Figure 5C). In order to facilitate the analysis of a concentration dependent inhibition of TeNT-Hc binding to gangliosides by antibodies, half-maximal binding of TeNT-Hc to gangliosides was calculated. The half-maximal binding of TeNT-Hc to gangliosides GM1a, GD3 and GT1b were 24.2 nM, 24.6 nM and 26.0 nM respectively (Figure 5A–C).

**Figure 6:**
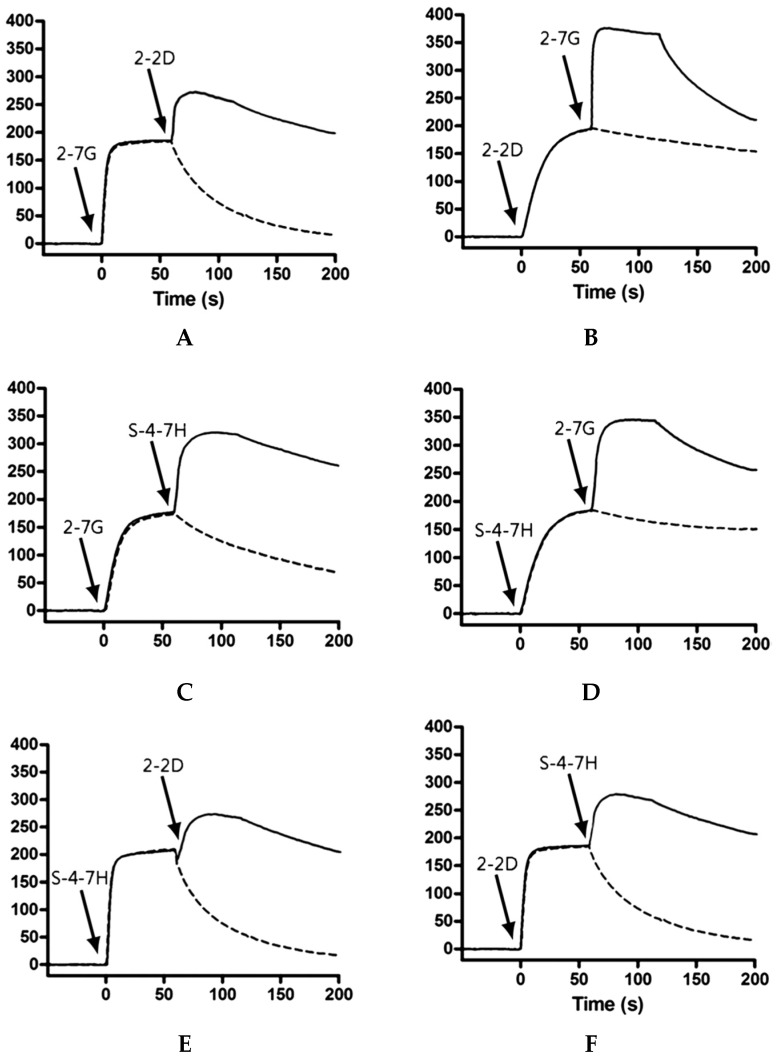
Anti-TeNT-Hc antibodies recognize overlapping and nonoverlapping epitopes. SPR co-injection experiments were used to determine whether pairs of antibodies could bind TeNT-Hc simultaneously. The sensorgrams of all of the possible paired combinations of 2-7G, 2-2D and S-4-7H in both orientations, are shown. Dashed lines represent injection of a single antibody followed by injection of buffer. Solid lines represent co-injections of the first antibody followed by injection of a second antibody. For all experiments, 80 μL of each antibody at a concentration 20× its KD value was injected over 10,098 RUs of immobilized TeNT-Hc at 40 μL/min. In general, 2-2D and S-4-7H appeared to partial inhibition in binding according to the equation above mentioned upon injection of the second species. Conversely, 2-7G appeared to bind a distinct, nonoverlapping epitope. When 2-7G injections were followed by 2-2D and S-4-7H injections, there was an approximate doubling of total signal with the second injection. However, this was not observed with the reverse injection combinations, because the relatively fast off-rates of 2-2D and S-4-7H compared with 2-7G resulted in very significant dissociation of these TeNT-Hc from the surface before equilibrium binding of 2-7G was reached. (**A**) SPR co-injection experiments of 2-7G followed by 2-2D; (**B**) SPR co-injection experiments of 2-2D followed by 2-7G; (**C**) SPR co-injection experiments of 2-7G followed by S-4-7H; (**D**) SPR co-injection experiments of S-4-7H followed by 2-7G; (**E**) SPR co-injection experiments of S-4-7H followed by 2-2D; (**F**) SPR co-injection experiments of 2-2D followed by S-4-7H.

Interestingly, 2-2D and S-4-7H antibodies both blocks binding of GD3 to the sialic acid binding site around R1226 in TeNT Hc and they could not be inhibited by each other according to a competitive binding ELISA. As we know, the advantages of a biosensor system include its ability to study molecular interactions in real time, with soluble proteins in their native state, unpurified and non-labelled. Biosensor technology instead of ELISA was chosen to determine whether 2-7G, 2-2D and S-4-7H compete in their binding to TeNT-Hc. We performed co-injection Biacore experiments with pairs of mAb (lgG), in both orientations, to determine whether antibodies could bind TeNT-Hc simultaneously (Figure 6). Of the paired combinations, only those involving 2-7G showed a significant increase in response consistent with theoretical Rmax values (~170–200 RUs) upon co-injection (Figure 6A,B). This suggests that 2-7G is free to bind TeNT-Hc when 2-2D or S-4-7H is bound and also indicates the 2-7G epitope is distinct and does not hinder binding of the other two mAbs. For 2-2D and S-4-7H only few changes in response were seen upon co-injection with theoretical Rmax values not reached, an indication that the mAbs were binding partly overlapping epitopes and hindering the binding of each other to TeNT-Hc (Figure 6C). Taken together, our Biacore based epitope mapping studies suggest 2-7G freely binds TeNT-Hc at a site that does not overlap with, or is sterically hindered by 2-2D or S-4-7H binding. 2-2D and S-4-7H bind at sites on TeNT-Hc that hinder freely accessible binding of the others, suggesting these two antibodies share bind epitopes in such close proximity to one another that it prevents unhindered interaction with sensor chip-immobilized TeNT-Hc.

### 2.7. In Vivo Toxin Neutralization

**Figure 7:**
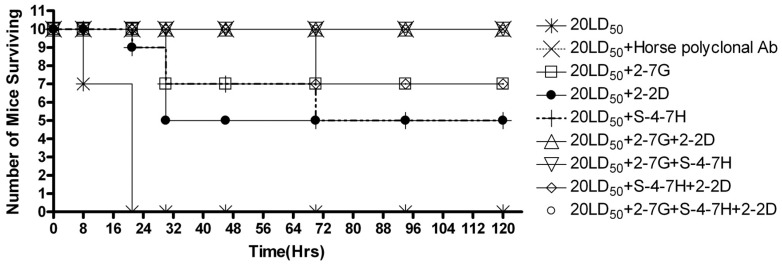
The biological activity of 2-7G, 2-2D, or S-4-7H, either individually or in combination, was determined using a tetanus toxin neutralization assay in mice. First, 20 µg total antibodies was mixed with 20 mouse LD_50_s of toxin and injected intraperitoneally (i.p.). Then, the time-to-death and the number of surviving mice were determined. No single mAb exhibited a significant protection against 20 LD_50_s, but could partly protect or prolong the time-to-death. All mice survived the challenge with 20 LD_50_s when administered the mixture of 2-7G, 2-2D, and S-4-7H IgG4.

A mouse assay was used to detect the toxin neutralization of 2-7G, 2-2D, and S-4-7H. Using the horse polyclonal anti-TeNT-Hc antibodies as a positive control, 20 µg of single antibody or antibody mixture was premixed with 20 LD_50_s TeNT and injected i.p. into the mice. The mice challenged with 20 LD_50_s TeNT all died within 24 h. Although no single antibody exhibited significant protection against 20 LD_50_s, 2-7G could protect 70% mice from death, and 2-2D or S-4-7H could prolong the time to death. All mice survived the challenge with 20 LD_50_s when administered the mixture of 2-7G, 2-2D, and S-4-7H (Figure 7), and the combination of the two antibodies could protect 70%–100% mice from death. The results indicated that 2-7G, 2-2D, and S-4-7H had a certain degree of protection against TeNT, whereas the combination of two or three antibodies had a greater potency of in vivo toxin neutralization. This may be due to the increase in the blockade of the toxin surface that binds to cellular receptors.

## 3. Discussion

The toxicity of tetanus toxin is among the most potent and lethal known toxins, with an estimated human lethal dose of less than 2.5 ng/kg. Tetanus is a fatal disease, resulting in an extremely high mortality rate between 40% and 78%, mainly due to respiratory failure [26,27]. Although tetanus is preventable by vaccination and post-exposure prophylaxis, the disease remains life-threatening, causing thousands of annual deaths worldwide in places in which the population is not extensively immunized [28].

The treatment for human tetanus has remained unchanged since the early 1900s by passively administering animal-derived anti-tetanus serum containing polyclonal immune immunoglobulins (PAb) together with supportive measures (e.g., an artificial respirator) to the patient. Several obstacles are faced both for the production and use of the therapeutic immune serum derived from animals. The major ones include adverse reactions due to the foreignness of the heterologous proteins to the recipient immune system, the limited supply of the immune serum, and the batch-to-batch variation of the antitoxin potency. In many developing countries, the current treatment consists of an intravenous infusion of equine antibody-based antitoxin, which can be associated with side effects, including hypersensitivity (e.g., serum sickness and anaphylaxis).

Next-generation antitoxins are likely to be derived from human monoclonal antibodies mapping different TeNT epitopes. Fully human neutralizing antibodies were deemed to be the most ideal and effective form of TeNT therapy. Phage display technology is a tractable, economical, and rapid method to obtain human antibodies via a high throughput. Theoretically, if the library is sufficiently large in diversity and size, most antibodies can be selected from it.

Numerous monoclonal antibodies have been developed against TeNT and tested for their neutralizing activity. Some monoclonal antibodies were generated against TeNT using mouse hybridoma technology [29,30,31], and some were selected from human phage libraries [32,33]. However, there are few existing reports describing selection from human immune libraries.

Antibodies with a high affinity are ideal candidates for clinical treatment. The affinities of the antibodies are correlated with the size of the repertoires. The larger the antibody library is, the higher the affinity of the antibodies that can be selected from it [34,35]. In our study, a human immunized scFv antibody phage display library of 1 × 10^8^ was used. 2-7G, 2-2D, and S-4-7H antibodies with a KD of 1.50 × 10^−10^ M, 7.82 × 10^−9^ M and 2.25 × 10^−9^ M were obtained. The three antibody clones can easily be associated with the antigen and are difficult to dissociate, which are essential characteristics for therapeutic antibodies.

The affinities of the three IgGs (2-7G, 2-2D and S-4-7H) increased by 2.9 to 10.1 times, compared with the scFv form. The antigen binding affinity of the IgG was significantly higher (lower KD) than for the corresponding scFv were also showed by James Marks’ group [36]. MAbs S25 and 3D12 that were selected from phage libraries against botulinum toxoid improved their affinities by 18.7 and 656 times from scFv to lgG form respectively [36]. Another example was mAb C10 which was selected from phage library against BoNT/A-Hc by our group [9]. The KD value with C10 changed from 7.81 × 10^−9^ M to 6.46 × 10^−11^ M, increased by 120 times from scFv to lgG form. After IgG construction the affinity of corresponding scFv may increase, decrease or remain unchanged. In the current study, 2-7G, 2-2D and S-4-7H scFvs were all maintain special binding to TeNT-Hc and neutralizing potency in vivo. The antigen binding affinity of the IgG was significantly higher than that of the corresponding scFv, because of an increase in the association rate constant (kon), and decrease in the dissociation rate constant (koff), like 2-7G and 2-2D. However, not the same as 2-7G and 2-2D, S-4-7H which significantly decreased in the koff increased the affinity by 10 times. This was also showed in scFv of 3D12 which significantly decreased in the koff by 4 times [36]. The affinity increased in scFv changed to lgG was also due to an increase in the stability of the molecule and hence an increase in the functional antibody concentration [36]. This was also found in scFv re-constructed to diabody or disulfide-stabilized scFv in our experiment. Furthermore, another reason may be that IgG was glycosylated and scFv from *E. coli* not, and glycosylation may affect antibody binding characteristics.

TeNT has AB structure-function properties: the A domain is a zinc metalloprotease, and the B domain encodes a translocation domain and *C*-terminal receptor-binding domain (TeNT-Hc). Earlier studies demonstrated that TeNT-Hc bound to gangliosides via two carbohydrate-binding sites termed the lactose-binding site (the “W” pocket) and the sialic acid-binding site (the “R” pocket) [37]. In this article, purified protective TeNT-Hc antigen was used as a selection antigen to screen neutralizing antibodies from the antibody phage display library. After three rounds of selection, more than 100 specific clones were obtained, and 12 clones were chosen based on the high OD values. Eight different sequences were identified from 12 clone sequences and three unique antibodies were mapped to different epitopes, whereas all others were mapped to the same epitope with the 2-7G. This may be due to the existence of a preponderant epitope on TeNT-Hc, which can bind to the phage antibodies with high affinity.

NGF-differentiated PC12 cells represent a widely used neuronal-like model system. After differentiation, they develop extended neuritis and an increased number of synaptic-like vesicles [38]. In the present study, when the antibodies were cultured with TeNT-Hc prior to being added to the NGF-differentiated PC12 cells, 2-7G, 2-2D, and S-4-7H could effectively inhibit the binding of TeNT-Hc to PC-12. Each of these three clones has a greater potency of toxin neutralization in vitro.

Previous studies have shown that GD3 bound the R pocket, and GM1a bound to the W pocket, whereas GT1b binds to both carbohydrate pockets of TeNT. Therefore, these gangliosides are useful tools for determining the roles of the W and R pockets in TeNT binding to neurons [37]. Our results indicate that the 2-7G inhibits TeNT-Hc binding to the receptor via the carbohydrate-binding sites of the W pocket, and 2-2D as well as S-4-7H inhibits TeNT-Hc binding to the receptor via the carbohydrate-binding sites of the R pocket. 

A dual-receptor model incorporating ganglioside receptors has been proposed to explain the distinct sites of action of TeNT [39]. These models attempt to explain the experimental observations that are largely inconsistent with a sole ganglioside receptor (e.g., the difference in the binding affinity measured in vivo and in vitro) [37]. Previous studies also demonstrated the simultaneous binding of two GT1b molecules to TeNT via the R and W binding sites, which was supported by the subsequent observation that GT1b can bind independently to the R or W pockets [37]. This suggests that the observed binding to these gangliosides represents the simultaneous occupancy of both the W and R pockets of TeNT by two molecules of GT1b. The TeNT-Hc bound cells with high affinity only in the presence of gangliosides that together, can bind both the R and W carbohydrate-binding pockets [37,40,41]. 

An interesting finding of this study was to test the synergistic neutralizing effect of the GT1b-binding inhibitory mAb. Cooperative binding is caused by conformational changes of the protein in which the binding of one antibody thermodynamically or entropically facilitates the binding of the next one. Such cooperative or synergistic effects have been reported for antibodies or scFvs binding to non-overlapping or independent epitopes of the tetanus toxin. Furthermore, another thing we concerned about was that when blocking specifically one carbohydrate-binding site of TeNT-Hc, the binding of GT1b to the other binding site was not visible in Figure 5C. GM1a contained Gal4-GalNAc3- moieties, while GD3 contained Sia7-Sia6- moieties, and GT1b contained those two moieties so as to it could be bind to “R” and “W” pocket binding site at the same time [37]. However, the structure of GT1b was not equal to the combination of GM1a and GD3. Occupation of two carbohydrate-binding pockets by gangliosides is required for high affinity binding of TeNT-Hc into PC12 Cells. Although TeNT-Hc did not bind to PPMP-treated PC12 (endogenous gangliosides of PC12 cells were depleted) cells preloaded with either GD3 or GM1a alone, TeNT-Hc bound PPMP-treated PC12 preloaded with a mixture of GD3/GM1a (1:1). TeNT-Hc also bound with high affinity PPMP-treated PC12 cells that were preloaded with GT1b. Interestingly, TeNT-Hc binding intensity in 2 mol of GT1b was higher level than that of GD3/GM1a (1 mol:1 mol). This showed that GT1b was more powerful than GD3/GM1a in promoting the combination of TeNT-Hc and PPMP-treated PC12 [37]. Therefore we presumed the greater the contribution, the greater the inhibitory effect of the combination. That may be why antibody alone could inhibit the combination of TeNT-Hc and GT1b from 78.6% to 82.3%. In our future research, GD3/GM1a will be instead of GT1b to investigate the ability of antibody-mediated inhibition of TeNT-Hc binding to GM1a. Another reason may be the spatial structure which played an important role in the combination of TeNT-Hc and GT1b. The crystal structure of ganglioside-TeNT-Hc complexes shows cross-linking between the GT1b and TeNT-HC. A single ganglioside could bind simultaneously to more than one TeNT molecule [42]. The inhibitory of one side of carbohydrate-binding pockets may affect the spatial structure of the cross-linking between GT1b and TeNT-HC, or even affect the combination of another GT1b to TeNT-HC. 

The W pocket is a groove formed by Trp1289, His1271, and Tyr1290 [42]. Trp1289 plays a dominant role in ganglioside interaction at the W pocket, thus the 2-7G inhibits TeNT-Hc binding to the receptor by recognizing an epitope that must encompass or lie near Trp1289. The R pocket consists of residues Asp1147, Arg1226, Asn1216, Asp1214, and Tyr1229, where the formation of a salt bridge between the carboxyl group of sialic acid and the guanidine group of Arg1226 is the most prominent feature [42]. We performed co-injection Biacore experiments with pairs of scFvs, to determine whether antibodies could bind TeNT-Hc simultaneously. For 2-2D and S-4-7H only few changes in response were seen upon co-injection with theoretical Rmax values not reached, an indication that the scFvs were binding partly overlapping epitopes and hindering the binding of each other to TeNT-Hc. Its importance of Arg1226 is highlighted by the drastic drop in toxicity to 1.4% and the almost complete loss of binding in the synaptosome assay upon mutation of R1226 to leucine or phenylalanine. Placing a bulky phenyl ring at the opening of the binding pocket, as implemented by TeNT-Gly1215Phe, reduced the toxicity to 15% in the mouse phrenic nerve (MPN) assay, the binding to 18% in the synaptosome assay compared to the wild-type values. The mutation interferes with H-bonding between D1214 and N1216 and sialic acid, and partially shields the central R1226 residue, according to molecular modeling analyses [40]. Accordingly, the 2-2D and S-4-7H epitopes map may encompass or lie near Arg1226 or Gly1215 respectively. In this study, 2-7G/2-2D and 2-7G/S-4-7H exhibits marked synergistic effects for the neutralization of TeNT-Hc binding to GT1b, while 2-2D/S-4-7H exhibits slightly synergistic effects with respect to neutralization.

The mixture of two or three antibodies was more effective than each single one for the neutralizing activities in vivo. Although the combination of 2-2D/S-4-7H and 2-7G could both protect 70% mice from death, the combination of 2-2D/S-4-7H exhibits slightly synergistic effects with respect to in vivo toxin neutralization than that of 2-7G. Any pair of mAbs that belonged to different carbohydrate-binding sites completely protected mice challenged with 20LD_50_s of toxin. The combination of 2-7G/2-2D and 2-7G/S-4-7H which belonged to different carbohydrate-binding sites exhibits marked more synergistic effects for the in vivo toxin neutralization than that of 2-2D/S-4-7H which belonged to the same carbohydrate-binding sites. All of the mice survived the challenge with 20 LD_50_s when given the mixture of the three antibodies. 

As we know, with a similar structure to BoNT-Hc, TeNT-Hc consisted of a two-receptor model incorporating ganglioside and protein receptors. Similar findings of neutralizing antibodies by James Marks’ group also shown that botulinum neurotoxins/A, can be potently neutralized by an oligoclonal Ab consisting of only three mAbs (MAbs S25, 3D12 and C25) [43,44]. MAbs S25 and C25 were selected from phage libraries constructed using mice immunized with BoNT/A-HC, while mAb 3D12 was selected from humans immunized with pentavalent botulinum toxoid. The affinities of those three antibodies were from 7.3 × 10^−^^8^ M to 1.1 × 10^−^^9^ M. Like 2-2D and S-4-7H in this study, the S25 and 3D12 epitopes map within the *C*-terminal subdomain, containing the putative sialo-ganglioside binding site. However, unlike 2-7G, C25 maps to a complex epitope that includes the majority of the Hc sequence, suggesting an epitope of adjacent *N*- and *C*-terminal subdomains. 50 micrograms of each single mAb prolonged the time to death but failed to protect mice challenged with 20 LD_50_s. Any pair of mAbs completely protected mice challenged with 100 LD_50_s of toxin. All mice receiving a mixture of all three mAbs (oligoclonal Ab) survived challenge with 500 LD_50_s of toxin [36]. 

The potential mechanism for potent tetanus toxin neutralization by oligoclonal Ab is the need to block multiple epitopes on the toxin binding domain surface that bind to cellular receptors. Broad interaction of the *C*-terminal subdomain with cellular receptors is consistent with the mechanism of Botulinum Neurotoxin [31]. Epitope mapping provides insight into why a single mAb cannot potently neutralize a toxin. Two spatially separated ganglioside binding sites have been observed in the co-crystal structure of the homologous tetanus toxin [42], which that gangliosides are functional dual receptors for TeNT. Potent toxin neutralization would require blockade of this broad surface, which could not be covered by a single Ab. Administration of all three mAbs may more potently neutralize toxin by blocking a larger proportion of the binding surface. Furthermore, antibodies that recognize different carbohydrate-binding pockets could exhibit higher synergistic toxin neutralization activities than those that recognize the same carbohydrate-binding pockets.

Each antibody appeared to inhibit the binding of TeNT-Hc to PC-12 cells and ganglioside in vitro, whereas a greater potency of neutralizing activities in vivo was observed only when two or three antibodies were combined. In our opinion, three reasons may explain this phenomenon: (1) it has been suggested that different factors (e.g., ganglioside concentration, GPI-anchored proteins, and pH) of the microenvironment play a major role in TeNT binding and intoxication of neurons; (2) it may be due to a difference between the PC-12 cells treated with NGF and the targeted nerve cells in vivo. The PC-12 cell line is established from a rat pheochromocytoma and has many properties in common with primary sympathetic neurons. Although PC-12 cells can differentiate both morphologically and biologically into neuronal cells, it is not the same with the neurons in vivo, including the expression of the TeNT receptors; and (3) it may be due to the sensitivity of the in vitro assay, which is much less than an in vivo neutralizing test. There may be still a few proteins that bind TeNT-Hc to the PC-12 cells, which cannot be observed by the naked eye. Therefore, the result of the in vivo study is more credible than the PC-12 model performed in vitro.

Additional antibodies that map different epitopes especially against the light chain should be further screened to exploit more potent neutralizations. We have noted that more recent studies on human-like recombinant antibodies confirmed the neutralizing role antibodies against the light chain of related toxin (Botulimum toxin) [45] and confirmed strong synergistic effect on combining the antibodies targeting different domains [46]. The antitoxins are no longer effective once the toxin is taken up into the neurons which happen faster than the identification of a botulism case since patients develop symptoms more than three days after intoxication [47]. Likewise, antibodies against the light chain of Tetanus Toxin may have a crucial role in treatment of tetanus toxin. 

The acquisition of neutralizing antibodies against TeNT exploited a novel and method to screen for human therapeutic antibodies via the human immunized scFv antibody phage display library.

## 4. Materials and Methods

### 4.1. Animals

Female, specific pathogen-free (SPF) BALB/c mice, weighing 16 g to 22 g each, were purchased from Beijing Vital River Laboratory Animal Technology Co. Ltd., Beijing, China. All animals were raised under humanitarian conditions. These mice with free access to food and water were obtained from the Laboratory Animal Center, Academy of Military Medical and Sciences. All efforts were made to minimize suffering. After injection, mice were followed for the five days to check for any signs of paralysis or death. Loss of righting reflex was used as the humane end point of the experiment. Mice were monitored three times a day for their condition and for the occurrence of end point. Mice that presented loss of righting reflex were humanely euthanized and sacrificed. All animal experiments used in this study were performed according to the protocols approved by the Institutional Animal Care and Use Committee of Beijing Institution of Biotechnology (Identification code for mice: IACUC of AMMS-08-2015-011; Date of approval: 9 December 2015). The final death total was determined five days after injection.

### 4.2. Human Antibody Phage Display Library

The human immune scFv antibody phage display library used in this study was constructed in our laboratory from the immunoglobulin genes of five volunteers who exhibited high antibodies titers against tetanus toxoid [14]. The details of the construction, the antibody diversity repertoire, and the other attributes of this phage library have been described elsewhere [14]. Briefly, genes encoding the V_H1_~V_H7_, Vκ_1_~Vκ_6_, and V_λ1_~V_λ10_ families of human immunoglobulin were amplified using 44 primer pairs. The primer set used for PCR amplification of antibody genes appends an SfiI restriction site at the 5′ end of the scFv cassette and a NotI site at its 3′ end.

The V_H_ and V_L_ amplicons were molecularly linked at random via a (Gly4Ser) 3 polynucleotide linker to generate a repertoire of VH-linker-VL or scFv. The human scFv (huscFv) sequences were cloned into pCANTAB5E phagemid vectors, and the recombinant phagemids were introduced into competent suppressor TG1 *E. coli* cells. Finally, the library was comprised of 1 × 10^8^ individual clones, and more than 95% of the phages in the library contained human scFv sequences in their genome.

### 4.3. Antigen Preparation

TeNT-Hc was expressed in *E. coli* BL21 (DE3) and purified, while the immunogenicity of TeNT-Hc was tested via the subcutaneous immunization of individual NIH mice as previously described [22]. A Bradford method kit (Tiangen Biotech, Beijing, China) was used to determine the concentration of the purified protein according to the manufacturer’s protocol.

### 4.4. Phage Preparation

The bacteria libraries from the human immunized scFv antibody library were diluted with blocking buffer (1× PBS containing 10% nonfat dry milk) and incubated at room temperature for 20 min. The diluted libraries were added to the 2× YT medium and the culture was incubated at 37 °C, 220 rpm to an OD600 of 0.5. M13KO7 helper phages (GE Healthcare, Waukesha, WI, USA) were added to the bacteria at an M.O.I. of 20:1 and left at 37 °C with no shaking for 30 min followed by gentle shaking at 150 rpm for another 30 min at 37 °C. The culture was then centrifuged, and the cell pellets were resuspended in 2TY-AG (100 µg/mL ampicillin, 1% glucose). After the addition of kanamycin to a final concentration of 50 µg/mL and IPTG (isopropyl thio-β-d-galactoride) to 0.1 mM, the bacteria were then cultured overnight at 30 °C, 200 rpm. The culture supernatant was purified and concentrated by PEG (20% polyethylene glycol 8000, 2.5 M NaCl) precipitations, and resuspended in PBS (pH 7.4).

### 4.5. Phage Display Biopanning

The immunotube (Nunc, Roskilde, Denmark) was coated with 200 nM TeNT-Hc or 200 nM pure active toxin (National Institutes for food and drug control, Beijing, China) in 0.05 M Na_2_CO_3_ buffer (pH 9.6) at 4 °C overnight. After rinsing twice with PBS, the immunotube was blocked with blocking buffer (1× PBS containing 3% nonfat dry milk) at room temperature for 2 h.

The pre-blocked antibody libraries were added to the immunotube and incubated at 37 °C for 90 min. The immunotube was then washed 10 times with PBST (PBS plus 0.1% Tween-20) followed by five rinses with PBS. The bound phages were eluted with 1.5 mL of 100 mM glycine-HCl (pH 2.2) for 15 min at room temperature and then neutralized with 1 M Tris-HCl to pH 7.4. The eluted phages were recovered by infecting the exponentially growing *E. coli* XL1-Blue. After culturing on Luria-Bertani (LB) medium-ATG (100 µg/mL ampicillin, 10 µg/mL tetracycline, 1% glucose) plates at 37 °C overnight, the XL1-Blue cells were scraped, and the phages were rescued by the helper phage M13KO7. The second and third panning rounds of the phage display scFv library were similar to that of the first round, except for the titers of the phage particles used and the concentrations of the coated antigen were lower than the first round. The titer of the unamplified third-round phage particles was determined on LB medium-CTG plates. 

### 4.6. Screening for Specific scFvs

After three rounds of panning, the phage clones were screened by an enzyme-linked immunosorbent assay (ELISA). The 96-well ELISA plate (Costar, Corning, NY, USA) was coated with 40 nM TeNT-Hc, and also coated with 130 nM *Yersinia pestis* capsular F1, 54 nM *Yersinia pestis* capsular V protein (F1 and V, purified in our lab) and 91 nM interferon-omega (IFN-ω, purified in our lab) individually as negative antigens.50 μL of phage particles at a concentration of approximately 8 μM in 3% Marvel™ PBS (pH 7.4) were added, and the binding of the phages to the immobilized antigen was performed at 37 °C for 2 h. The unbound phages were removed by washing extensively with PBST. The plate wells were detected by horseradish peroxidase (HRP)-conjugated anti-M13 antibody (GE Healthcare), followed by the addition of peroxidase substrate, o-phenylenediamine dihydrochloride (OPD). 2 M H2SO4 was used to stop the reaction, and the results were monitored at OD492/630 with a microplate reader (Multiskin MK3, Shanghai Labsystems, Shanghai, China). The particular clones that were bound to TeNT-Hc were selected, and the corresponding phage plasmids were sequenced. 

### 4.7. Subcloning and Expression of scFvs

The genes of immunopositive scFvs were cloned into the expression vector kil-SN through the restriction enzyme sites, SfiI and NotI. The kil-SN vector was reconstructed on the basis of pET22b (+) (Novagen, Madison, WI, USA), which contained the colicin release gene kil sequence, signal peptide pelB, E-tag, and 6× His. The recombinant plasmids were then transformed into the *E. coli* strains KS1000 (DE3) and cultured at 37 °C at 220 rpm to an OD_600_ of 0.5. After adding IPTG to a concentration of 0.005 mM, the culture was incubated at 30 °C and 220 rpm overnight for the detection of antibody expression. In the presence of kil proteins, the scFv fragment can be easily released from the periplasm into the growth medium. The supernatants containing scFv were harvested, and antibodies were purified with a HisTrap FF crude 1-mL column (GE Healthcare).

### 4.8. Epitope Mapping

A competitive binding ELISA was used to search for specific antibodies mapped to different epitopes of TeNT-Hc. After blocking, 50 µL of scFv at a concentration of approximately 8 μM was added to the plate coated with TeNT-Hc and incubated for 1 h [48]. After washing, the supernatants containing phage-scFvs were then added and incubated for 1 h. An HRP-conjugated anti-M13 antibody was used to detect antigen recognition, and the results were detected as above. The wells, which the *E. coli*-expressed scFvs were not added to, were used as the negative controls. If two detected antibodies mapped to the same or similar epitope, inhibition would be detected. The inhibition efficiency (IE) was counted using the following formula: IE (%) = (1 − OD492/630 of experimental well/OD492/630 of negative control well) 100%. IE ≥ 50% was considered an inhibition (+), 20% < IE < 50% was considered partial inhibition, and IE ≤ 20% was considered a non-inhibition.

### 4.9. ScFv SPR Kinetic Measurements

Antibody affinity was determined by surface plasmon resonance spectroscopy using a BIAcore T200 instrument (GE Healthcare-Biacore, Uppsala, Sweden). ScFv or lgG was coated at a maximum of 3500 RU (7000 RU for lgG) on a CM5 chip (BR100012, GE Healthcare-Biacore) via amine coupling according to the manufacturer’s instructions. A flow rate of 20 µL/min was maintained during each measurement and volumes of 100 µL of activated TeNT (125 nM to 0.1 nM) dissolved in HBS-EP buffer (BR100188, GE Healthcare-Biacore) were tested. After each dilution, the chip was regenerated with 1.5 µM glycine buffer (BR100354, GE Healthcare-Biacore) injected for 30 s at 10 µL/min. Affinities were calculated using the BIAevaluation software (GE Healthcare-Biacore) according to the Langmuir 1:1 adsorption model (except for 2-7G-lgG using bivalent model) and were verified by internal consistency test [49]. The number of technical replicates was equal to 3 and mean +/− standard deviations of Kon, Koff and KD were given.

### 4.10. Competition Studies Using SPR Analysis

We used Biacore co-injection experiments to determine whether our mAbs could bind unique, nonoverlapping epitopes on TeNT-Hc. Briefly, 80 μL of the first mAb diluted in HBS-EP buffer to a concentration of 20× its KD value was injected over 10,098 RUs of immobilized TeNT-Hc at 40 μL/min. Following injection of the first mAb, buffer or a second mAb (80 μL total volume, at 20× KD) was injected at 40 μL/min over the TeNT-Hc surface already saturated with the first mAb. Data were collected on all possible paired combinations of 2-7G, 2-2D, and S-4-7H, in both orientations (i.e., each mAb acted as the first and second mAb). For every mAb the percentage of binding to a specified amount of TeNT-Hc was determined. The Maximal RU mAb is determined by the highest response obtained while the first Ab binding to the immobilized TeNT-Hc. The percentage of binding of a certain mAb to TeNT-Hc was expressed relative to the maximal binding as obtained with any of the presenting mAb according to the following equation:

$$\frac{\frac{RU\;mAb}{RU\;TeNT-Hc}\;}{\frac{Maximal\;RU\;mAb}{RU\;TeNT-Hc}}\times 100\%$$

A percentage of binding of the second mAb, in relation to the TeNT-Hc presenting first mAb, of <10% of the maximal binding was defined as inhibition in binding. Binding of the second mAb of <50% and >10% of the maximal binding was arbitrarily defined as partial inhibition in binding. A binding of >50% of the maximal binding was defined as concurrent binding [50].

### 4.11. IgG4 Construction

The heavy and light chain expression vectors were used to cotransfect FreeStyle^TM^ 293-F cells (Invitrogen, Carlsbad, CA, USA) for instantaneous expression as a previously described procedure [9]. Supernatants containing IgG4 were collected and purified with the use of a HiTrap protein An FF 1-mL column (GE Healthcare).

V_H_ genes of scFvs were amplified using PCR from the *E. coli* expression vector kil-SN-scFv with the primer pairs CAGTGTGAAGTTCAACTG GTTCAAAGTGGTG and GTTCAGCTAGCGCTCGACACGGTCACCAGAGTGC. DNA digested with NheI was ligated into plasmid H293 (constructed by our lab, containing the CH domain of IgG4), which was digested with BstZ17I and NheI. The clones containing the correct V_H_ were identified by DNA sequencing. The V_λ_ genes of scFvs were amplified from the *E. coli* expression vectors kil-SN-scFv with the primer pairs CCTGGGCCAGC TACGAACTGA CCCAGCCG, and CATTAAGCTTGGTGCCACCGCCAAACAC. DNA, digested with HindIII, was ligated into plasmid L293-C_λ_ (constructed by our lab, containing the C_λ_ domain), which was digested with EcoRV and HindIII, and the clones containing the correct V_λ_ were identified via DNA sequencing. 

### 4.12. In Vitro Inhibition of PC-12 Cell Binding

The rat pheochromocytoma cell line (PC12) was cultured as described previously [51]. For crosslinking experiments, cells were seeded in 12-well plates (Costar-Corning, Cambridge, MA, USA) at a density of 25,000 cells/well. After 24 h, the medium was supplemented with 75 ng/mL nerve growth factor (NGF, produced by our lab). NGF-treated rat PC12 were prepared and fixed with 4% (*w*/*v*) paraformaldehyde in 100 mM sodium phosphate buffer (pH 7.4) for 30 min at room temperature. The antigenicity of certain surface receptors was not affected by this fixation procedure. Cells were incubated with 40 nM TeNT-Hc and mixed with or without the purified IgG4 antibodies (the final concentration was 40 nM) for 1 h. Mouse polyclonal anti-TeNT-Hc antibodies (produced in our lab) were used as the positive controls. Bovine serum albumin (BSA) was used as negative controls. The inhibition efficiency of the binding between TeNT-Hc and PC-12 cells was measured with the mouse polyclonal anti- TeNT antibodies (1:5000, Zhongshan Goldenbridge, Beijing, China) followed by FITC-labeled anti-mouse IgG antibodies (1:5000, Zhongshan Goldenbridge, China). Evan’s blue dye was used to stain the nuclei. The fluorescence was observed by an inverted Epi-fluorescence microscope (Eclipse TS300, Nikon, Amsterdam, Netherlands).

### 4.13. Assessment of mAb Inhibitory Activity on the TeNT Binding to Ganglioside

The ability of the mAbs to inhibit the binding of TeNT to ganglioside GT1b/GM1a/GD3 was assessed by a modification of a previously described procedure [31,52]. Briefly, a serial concentrations of TeNT-Hc (expressed by our laboratory, from 6 nM to 400 nM) was chosen for use in the assay, and incubated with an equal volume of anti-TeNT mAb (66.7 nM) for 2 h at room temperature. Microtiter ELISA plates were coated with ganglioside GT1b/GM1a/GD3 (Sigma, Saint Louis, MO, USA) (10 μg/mL in methanol, 100 μL/well) and the plates were left at room temperature overnight to allow for the evaporation of methanol. The plates were then blocked with 1% BSA/PBS for 2 h at room temperature. After washing four times with PBS-Tween 20, 100 μL of each TeNT-Hc/antibody mixture (preincubated for 2–3 h) was added to the wells and incubated for 2 h. The plates were washed four times and then incubated 2 h with HRP-conjugated human anti TeNT polyclonal antibodies. Following the addition of the TMB substrate solution and then adding the stop solution, the ODs were measured at 450 nm by a multiscan ELISA reader. The half-maximal binding of TeNT-Hc to gangliosides GT1b/GM1a/GD3 were calculated.

### 4.14. In Vivo Toxin Neutralization

In vivo toxin neutralization was studied with the help of a mouse assay in which the toxin and 20 µg of the antibodies were premixed and injected intraperitoneally (i.p.). TeNT neurotoxin was purchased from National Institutes for food and drug control, China. The protein content was 5 mg/mL (33.3 μM) with a specific toxicity in mice of 2 × 10^7^ LD50/mg protein. Every ten mice were classified in one group. The time-to-death and the numbers of surviving mice were determined. Using the horse polyclonal anti-TeNT-Hc antibodies showed 2000 IU/mL (Zhongshan Goldenbridge, Beijing, China) as a positive control. 20 µg of the appropriate IgG4 was added to 20 mouse LD50s of TeNT neurotoxin to a total volume of 0.5 mL of PBS and incubated at 37 °C for 30 min. For the Ab pairs, 10 µg of each Ab was added. For a combination of the three Abs, 6.6 µg of each Ab was added. The mixture was then i.p. injected into female Balb/c mice (16–22 g). Mice were studied in nine groups and were observed three times a day. The final death total was determined five days after injection.

## Figures and Tables

**Table 1:** Competitive binding ELISA between TeNT-Hc specific antibodies.

*E. coli*-Expression scFvs	Inhibition Efficiency of Phage-scFvs, %							
	2-1B	2-2D	3-6C	2-7G	5-1A	5-5E	S-1-1H	S-4-7H
2-1B	82	11	56	79	72	67	53	8
2-2D	12	76	18	4	9	9	5	11
3-6C	75	16	70	75	66	76	57	0
2-7G	83	6	82	85	78	79	87	3
5-1A	67	3	65	71	79	71	72	2
5-5E	58	9	71	77	69	77	59	7
S-1-1H	61	4	63	79	66	68	81	14
S-4-7H	4	15	0	2	2	6	9	74

**Table 2:** Association (kon) and dissociation (koff) rate constants and equilibrium dissociation constants (KD) for scFvs binding to TeNT (±S.D.).

scFv		TeNT	
	KD (M) (×10^−9^)	kon (M^−1^·S^−1^) (×10^5^)	koff (S^−1^) (×10^−4^)
S-4-7H	2.25 (±0.11)	1.91 (±0.05)	4.29 (±0.09)
2-2D	7.82 (±0.43)	0.61 (±0.12)	4.78 (±0.20)
2-7G	0.15 (±0.04)	2.52 (±0.07)	0.38 (±0.06)

**Table 3:** Families of the Light Chain and the Heavy Chain and the CDR3-Sequences of the scFvs Isolated from the Phage-Displayed Library.

Clone	Light Chain Family	VL CDR3	Heavy Chain Family	VH CDR3
2-7G	K3	HQYGSLPRT	H3	RRGANYYYGMDV
2-2D	K3	QQRTSWPPA	H3	ENHDSSGYFSRLSFDV
S-4-7H	L1	ATWGDALNGPV	H6	LGGGRYSYGYIPYYYYMDV

**Table 4:** Association (kon) and dissociation (koff) rate constants and equilibrium dissociation constants (KD) for lgGs binding to TeNT (±S.D.).

lgG		TeNT	
	KD (M) (×10^−10^)	kon (M^−1^·S^−1^) (×10^5^)	koff (S^−1^) (×10^−4^)
S-4-7H	3.57 (±0.12)	1.51 (±0.10)	0.54 (±0.06)
2-2D	11.16 (±2.21)	2.47 (±0.17)	2.76 (±0.20)
2-7G	0.52 (±0.08)	4.69 (±0.17)	0.24 (±0.01)

## Supplementary Materials

The following are available online at www.mdpi.com/2072-6651/8/9/266/s1. Table S1: Families of the heavy Chain and the CDR-Sequences of the scFvs isolated from the Phage-Displayed Library. Table S2: Families of the light Chain and the CDR-Sequences of the scFvs isolated from the Phage-Displayed Library. Figure S1: SDS-PAGE analysis of recombinant Kil-SN-scFv in *E. coli* BL21. M: Marker; Lane 1: supernatant of Kil-SN Induced with IPTG; Lane 2–9: supernatant of Kil-SN-scFv Induced with IPTG, from left to right: 2-1B, 2-2D, 2-7G, 3-6C, 5-1A, 5-5E, S-1-1H, S-4-7H. Figure S2: SDS-PAGE analyses of the His-scFv protein purified using Ni-NTA agarose column. M: Marker; Lane 1–8: purified His-scFv, from left to right: 2-1B, 2-2D, 2-7G, 3-6C, 5-1A, 5-5E, S-1-1H, S-4-7H. Figure S3: ELISA characterization of purified scFvs binding to different proteins. Assays were performed by immobilizing TeNT-Hc, F1, V, IFN-ω, and BSA-coated on a polystyrene plate. Phage-scFvs derived from the library that were reactive with the coated antigen, were detected with a 1:5000 dilution of horseradish peroxidase (HRP)–conjugated anti-His antibody. The results of the assay are shown as the absorbance at 492 nm/630 nm. Assays were performed in triplicate, and the range is shown.

## Abbreviations

The following abbreviations are used in this manuscript:

TeNT	Tetanus neurotoxin
TeNT-Hc	Hc fragments of TeNT
LD50s	50% of the lethal dose
MNT	neonatal tetanus
PAb	polyclonal antibodies
scFv	single-chain variable fragment
TIG	human Tetanus Immunoglobulin
Fab	antigen-binding fragment
“W” pocket	lactose-binding site
“R” pocket	sialic acid-binding site
IPTG	isopropyl thio-β-d-galactoride
PEG	20% polyethylene glycol 8000
PBST	PBS plus 0.1% Tween-20
ELISA	enzyme-linked immunosorbent assay
F1	*Yersinia pestis* capsular F1 protein
V	*Yersinia pestis* capsular V protein
IFN-ω	interferon-omega
HRP	horseradish peroxidase
OPD	o-phenylenediamine dihydrochloride
IE	inhibition efficiency
PC12	rat pheochromocytoma cell line
NGF	nerve growth factor
BSA	Bovine serum albumin
