# Supplementary material for: Tetanus Neurotoxin Neutralizing Antibodies Screened from a Human Immune scFv Antibody Phage Display Library
Source: Toxins (Basel). 2016 Sep 11;8(9):266. doi: 10.3390/toxins8090266 (PMC5037492; doi:10.3390/toxins8090266)
Supplement: Supplementary file 1 [file toxins-08-00266-s001.pdf]

Supplementary Materials: Tetanus Neurotoxin Neutralizing Antibodies Screened from a Human Immune scFv Antibody Phage Display Library

Han Wang, Rui Yu, Ting Fang, Ting Yu, Xiangyang Chi, Xiaopeng Zhang, Shuling Liu, Ling Fu, Changming Yu and Wei Chen

1. Families of the Heavy Chain and Light Chain the CDR-Sequences of the scFvs Isolated from the Phage-Displayed Library

Table S1. Families of the heavy Chain and the CDR-Sequences of the scFvs isolated from the Phage-Displayed Library.

Heavy Chain Family	Clone	VH		
		CDR1	CDR2	CDR3
VH2	2-1B	TSGVGVG	LIYWNDDKRYSPSLKS	ARDTSGKYSDYRTATYGYSMDY
VH3	2-2D	DYGMS	GINWNGGSTGYADSVKG	ENHDSSGYFSRLSFDV
VH3	2-7E	DYGMS	GINWNGGSTGYADSVKG	ENHDSSGYFSRLSFDV
VH3	2-7G	SNYMS	LISWDGGSTYYADSVKG	RRGANYYYGMDV
VH3	3-6C	SYGMH	VISYDGSNKYYADSVKG	ATFCDYTDYLGRCEALES
VH5	5-1A	SYWIS	RIDPSDSYTNYSPSFQG	VKRGGDNGWGAFDV
VH1	5-5E	SSAVQ	WIVVGSGNTNYAQKFQE	ARVPETTVTTGPLLYYYYGMDV
VH2	S-1-1H	SSYGMH	RIDWDDDKFYSTSLKT	AEAGAAAWATGANALDA
VH2	S-3-2D	SSYGMH	RIDWDDDKFYSTSLKT	AEAGAAAWATGANALDA
VH6	S-1-7C	SNSAAWN	RTYYRSKWYNDYAVSVKS	LGGGRYSYGYIPYYYYMDV
VH5	S-4-6C	SSYGMH	RIDWDDDKFYSTSLKT	AEAGAAAWATGANALDA
VH6	S-4-7H	SNSAAWN	RTYYRSKWYNDYAVSVKS	LGGGRYSYGYIPYYYYMDV

Table S2. Families of the light Chain and the CDR-Sequences of the scFvs isolated from the Phage-Displayed Library.

Light Chain Family	Clone	VL		
		CDR1	CDR2	CDR3
VKI	2-1B	RASQSISSWLA	DASSLES	QQYNSYS
VK3	2-2D	RASQSVSSSYLA	GASSRAT	QQRTSWPPA
VK3	2-7E	RASQSVSSSYLA	GASSRAT	QQRTSWPPA
VK3	2-7G	RASQSVSSNLA	GASTRAT	HQYGSLPRT
VKI	3-6C	WASQGISSYLA	YASSLQS	QQYYSTP
VKI	5-1A	RASQGISSWLA	AASSLQS	QQANSFP
VK3	5-5E	RASQSVSSYLA	DASNRAT	QQRSNWP
VK3	S-1-1H	RASQSVSSSYLA	GASSRAT	QQYGSSP
VK3	S-3-2D	RASQSVSSSYLA	GASSRAT	QQYGSSP
Vλ1	S-1-7C	SGSSSNIGNNAVN	YDDLLPS	ATWGDALNGPV
VKI	S-4-6C	RASQSVSSSYLA	GASSRAT	QQYGSSP
Vλ1	S-4-7H	SGSSSNIGNNAVN	YDDLLPS	ATWGDALNGPV

2. SDS-PAGE of the scFv Expressed and Isolated from *E. coli*

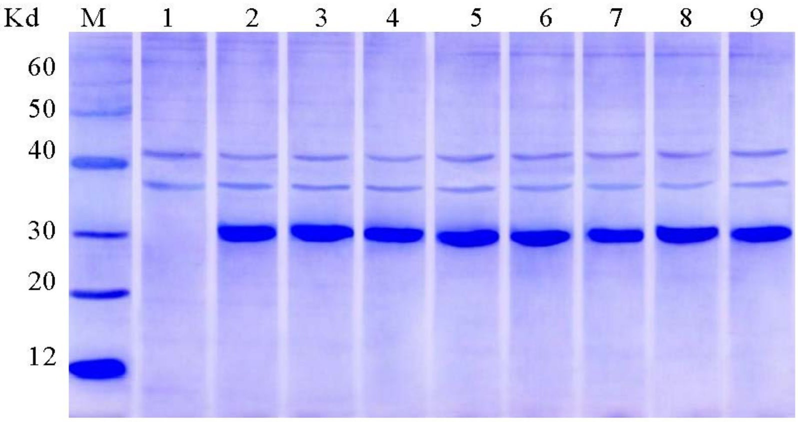

Figure S1. SDS-PAGE analysis of recombinant Kil-SN-scFv in *E. coli* BL21. M: Marker; Lane 1: supernatant of Kil-SN induced with IPTG; Lane 2–9: supernatant of Kil-SN-scFv induced with IPTG, from left to right: 2-1B, 2-2D, 2-7G, 3-6C, 5-1A, 5-5E, S-1-1H, S-4-7H.

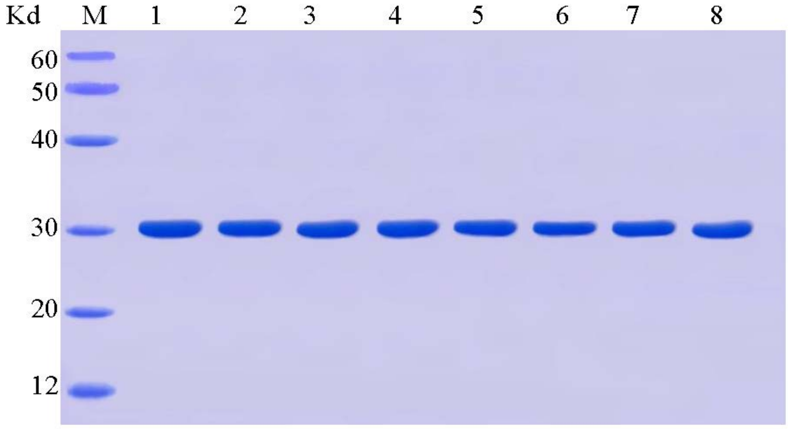

Figure S2. SDS-PAGE analyses of the His-scFv protein purified using Ni-NTA agarose column. M: Marker; Lane 1–8: purified His-scFv, from left to right: 2-1B, 2-2D, 2-7G, 3-6C, 5-1A, 5-5E, S-1-1H, S-4-7H.

3. ELISA Characterization of Purified scFvs Binding to Different Proteins

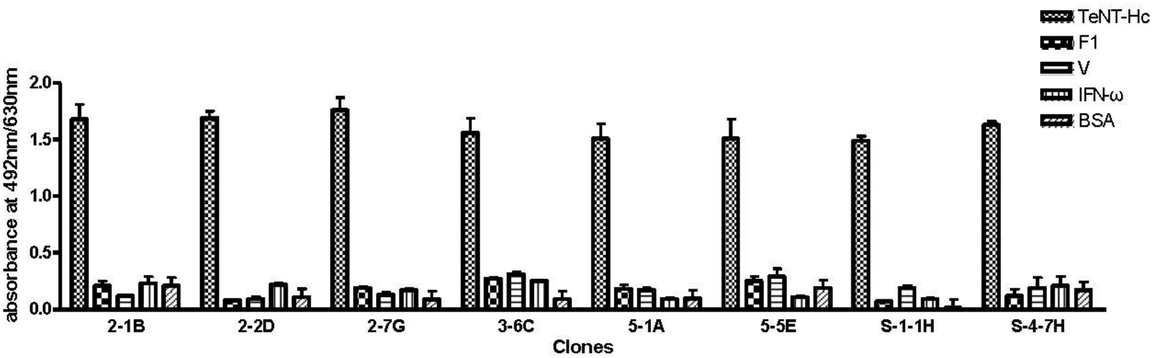

Figure S3. ELISA characterization of purified scFvs binding to different proteins. Assays were performed by immobilizing TeNT-Hc, F1, V, IFN-ω, and BSA-coated on a polystyrene plate. Purified scFvs that were reactive with the coated antigen, were detected with a 1:5000 dilution of horseradish peroxidase (HRP)–conjugated anti-His antibody. The results of the assay are shown as the absorbance at 492 nm/630 nm. Assays were performed in triplicate, and the range is shown.
